# Procedural sedation and analgesia for respiratory-gated MR-HIFU in the liver: a feasibility study

**DOI:** 10.1186/s40349-016-0063-x

**Published:** 2016-07-29

**Authors:** Johanna M. M. van Breugel, Joost W. Wijlemans, Hermanus H. B. Vaessen, Martijn de Greef, Chrit T. W. Moonen, Maurice A. A. J. van den Bosch, Mario G. Ries

**Affiliations:** 1 Division of Imaging, University Medical Center Utrecht, Utrecht, The Netherlands; 2 Division of Anesthesiology, University Medical Center Utrecht, Utrecht, The Netherlands; 3 Department of Radiology, University Medical Center Utrecht, Postbox: 85500, Heidelberglaan 100, 3508 GA Utrecht, The Netherlands

**Keywords:** Procedural sedation and analgesia, General anesthesia, MR-HIFU, Liver, Thermal ablation

## Abstract

**Background:**

Previous studies demonstrated both pre-clinically and clinically the feasibility of magnetic resonance-guided high-intensity focused ultrasound (MR-HIFU) ablations in the liver. To overcome the associated problem of respiratory motion of the ablation area, general anesthesia (GA) and mechanical ventilation was used in conjunction with either respiratory-gated energy delivery or energy delivery during induced apnea. However, clinical procedures requiring GA are generally associated with increased mortality, morbidity, and complication rate compared to procedural sedation and analgesia (PSA). Furthermore, PSA is associated with faster recovery and an increased eligibility for non- and mini-invasive interventions.

**Methods:**

In this study, we investigate both in an animal model and on a small patient group the kinetics of the diaphragm during free-breathing, when a tailored remifentanil/propofol-based PSA protocol inducing partial respiratory depression is used. Subsequently, we demonstrate in an animal study the compatibility of the resulting respiratory pattern of the PSA protocol with a gated HIFU ablation in the liver by direct comparison with gated ablations conducted under GA. Wilcoxon signed-rank tests were performed for statistical analysis of non-perfused and necrosed tissue volumes. Duty cycles (ratio or percentage of the breathing cycle with the diaphragm in its resting position, such that acoustic energy delivery with MR-HIFU was allowed) were statistically compared for both GA and PSA using student’s *t* tests.

**Results:**

In both animal and human experiments, the breathing frequency was decreased below 9/min, while maintaining stable vital functions. Furthermore an end-exhalation resting phase was induced by this PSA protocol during which the diaphragm is virtually immobile. Median non-perfused volumes, non-viable volumes based on NADH staining, and duty cycles were larger under PSA than under GA or equal.

**Conclusions:**

We conclude that MR-HIFU ablations of the liver under PSA are feasible and potentially increase the non-invasive nature of this type of intervention.

## Background

High-intensity focused ultrasound (HIFU) ablation of liver tumors was one of the first HIFU applications under investigation [1]; even after several decades of methodological refinement, HIFU ablation in the liver faces still several challenges, which hamper widespread clinical adoption and remains a topic of research [2–6]. The thoracic cage and the lung partially obstruct acoustic access to the tumor, thereby limiting the locations directly accessible with extracorporeal HIFU-transducers [7, 8]. Furthermore, the high perfusion of the liver requires high acoustic intensities in the focus during a sonication for an efficient energy deposition to overcome this so-called heat-sink effect [9, 10], which in-turn limits the achievable ablation volume per sonication. Finally, respiratory motion causes a continuous displacement of the target lesion.

Two techniques for guidance of HIFU-treatments are widely used, which are ultrasound [11, 12] and magnetic resonance imaging. With respect to the previously mentioned challenges in the liver, both methods have their specific advantages and disadvantages. In this paper, we focus on magnetic resonance guided HIFU (MR-HIFU). MR-HIFU applies high-intensity sound waves transcutaneous into the body for thermal tissue ablation and is a fully non-invasive therapeutic modality. The excellent anatomical contrast of the associated MRI is thereby used for localization and delineation of the lesion and the possibility of accurate real-time temperature monitoring allows guidance during tissue ablation. As a consequence, MR-HIFU is meanwhile a well-established treatment option for uterine fibroids and is currently investigated for, e.g., bone tumors [13], breast cancer [14, 15], prostate cancer [16], and liver cancer [10, 17].

In MR-guided liver treatments an additional challenge is the continuous movement of the target due to the respiratory cycle that requires motion compensation strategies for both MR thermometry and acoustic energy deposition. For the latter, several strategies have been suggested to address respiratory induced liver motion, ranging from induced apnea, over gated approaches, to dynamic beam-steering [3, 18, 19].

Similarly, real-time PRF-thermometry (proton resonance frequency-thermometry) for therapy guidance has been adapted to respiratory motion with multi-baseline phase corrections, referenceless PRF-thermometry approaches, hybrid correction schemes, and gated MR-thermometry [20–23].

Although MRI guidance has the advantage of real-time temperature mapping, to date, very few studies have combined MR guidance with the abovementioned HIFU strategies on clinical equipment into a clinically feasible treatment protocol [24]. Animal studies focused mainly on technical developments, while clinical studies mostly used ultrasound guidance, which currently lacks real-time temperature monitoring. The few liver tumor treatments with MR-HIFU that have been reported all used controlled apnea to mitigate respiratory problems [24–26]. More recently, our group studied MR-HIFU of the liver in a porcine model and demonstrated the feasibility of creating a confluent ablation volume using clinical HIFU equipment, which is modified to allow respiratory gating [10]. The ablations were performed under general anesthesia (GA) using mechanical ventilation in combination with an MR pencil beam navigator on the diaphragm to allow for respiratory-triggered acquisition of the anatomical scans and thermometry and most importantly for respiratory-gated acoustic energy deposition. The latter assures that no energy is deposited outside the target region if the diaphragm—and thereby the liver—is displaced from its resting position due to respiration. By using mechanical ventilation, the frequency of inhalation and exhalation and the duration of the resting phase can be chosen within the boundary of physiologically safe parameters.

Alternatively, another particular form of ventilation that can be used under general anesthesia is high frequency jet ventilation, which is able to deliver jets at rates of 60–100 min^−1^ [27]. The advantage of this technique is the small tidal volume leading to little respiratory motion (3 mm) [28, 29]. However, there are several disadvantages to this technique, such as its invasive nature due to the need for total muscle relaxation and the insertion of an endotracheal tube; carbon dioxide monitoring is difficult and requires, e.g., cessation of respiration for side-stream sampling or gas sampling from an arterial line; emergent re-intubation with a common endotracheal tube was needed in 10 % of all study subjects as laryngeal mask ventilation was insufficient and oxygen saturation dropped below 70 %, and several risks are present while using this technique such as regurgitation (gastroesophageal reflux), severe bleeding, and pulmonary barotrauma [27, 30]. Concerns mentioned by the authors of two recent papers include inability to treat tumors in the liver dome area as they may be obscured by the lung, the non-existence of an MR-compatible device to induce high frequency jet ventilation, and the requirement of a specialized anesthetic team [31–33].

In summary, all aforementioned approaches, including induced apnea, mechanical ventilation with gating strategies, or high-frequency jet-ventilation require essentially GA over the entire duration of the intervention. Unfortunately, GA is frequently associated with mortality, morbidity, and complications [34], in particular with elderly and more fragile patients in advanced stage of disease [35, 36]. Of all deaths, 46.6 % were associated with an anesthesia overdose, 42.5 % with adverse effects, and 3.6 % with pregnancy/obstetric care. Known complications are impaired cardiovascular function, postoperative cognitive dysfunction in the elderly, cardiac arrest (sometimes with fatal outcome), hypotension and bradycardia, peripheral neuropathies, skin burns, dental damage, acute myocardial infarction, and irreversible cerebral damage [37–40]. An increase in death rates was observed in patients with relevant comorbidities, which is not an unlikely case in HIFU patients, and according to German data, the proportion of patients older than 65 who had to undergo surgery rose from 38.8 % (*n* = 4.7 million) to 40.9 % (*n* = 5.9 million) from 2005 to 2009 [36]. In other words, anesthesia-associated mortality has risen again in purely numerical terms due to an increase in the proportion of elderly—often multimorbid—undergoing surgery. Elderly people with comorbidities are one of the target groups of MR-HIFU due to its non-invasive nature. Since MR-HIFU interventions are generally lengthy (>2–3 h), the need for GA over these long durations reduces the non-invasive aspect of MR-HIFU, which represents one of its major advantages.

A potential way to overcome this shortcoming is the use of procedural sedation and analgesia (PSA) [41] instead of GA. In comparison, PSA is known to cause fewer complications and has a better compatibility with other flanking oncological interventions, such as chemotherapy or surgery. Respiratory arrest and airway obstruction should be carefully checked for throughout the procedure as these are two possible complications of PSA, although not commonly encountered. PSA ususally has minimal influence on cardiovascular function [42].

Points of comparison where PSA shows more favorable characteristics than GA are, e.g., less requirement for intervention of the airways during a procedure, the ability to maintain spontaneous ventilation during a treatment, and the ability to maintain good cardiovascular function [41]. Furthermore, recovery is faster after PSA leading to the possibility of ambulatory treatment. Therefore, the burden and risks of PSA for patients is much less than for GA. Moreover, patients with severe disease may not be eligible for, e.g., MR-HIFU performed under GA but can be treated under PSA.

On the other hand, the principal shortcoming of PSA as a replacement for GA in combination with mechanical ventilation is that the patient is essentially free-breathing during the entire duration of the intervention. While the respiratory breathing pattern under mechanical ventilation displays in general a brisk and short inhalation-exhalation event, followed by a lengthy period of complete standstill of the diaphragm, free-breathing results in much longer inhalation-exhalation events, with a smoothed out motion pattern of the diaphragm, which is also frequently subject to drifts and a-periodic events [43]. As a consequence, the achievable duty cycle for gated HIFU energy delivery under free-breathing is substantially more unfavorable compared to mechanical ventilation.

A potential way to overcome this disadvantage is to manipulate the kinetics of the diaphragm during free-breathing by the appropriate medication. One of the oldest known families of drugs introducing a partial respiratory depression are opioids. However, there are several types of opioid receptors in the human body, for which each type of opioid displays different affinities and interactions, which in turn leads to unique modifications of the respiratory cycle [44, 45]. Two of the most frequently used opioids in clinical anesthesia are fentanyl and its N-4 thienyl derivative, sufentanil, which are consequently well understood with respect to their effect on the respiratory system [46].

In the scope of this study, we investigate in a porcine model if a remifentanil-based PSA protocol leads to a slow and regular respiratory pattern, which allows an effective and reproducible respiratory-gated MR-HIFU energy deposition in the liver. Since in addition to the required partial respiratory depression a sedative effect is highly desirable, remifentanil is thereby combined with the hypnotic/amnestic agent propofol, which represents one of the most wide-spread used drugs for the induction and maintenance of general anesthesia or sedation [47]. The study compares directly MR-HIFU ablations under PSA and under GA with respect to achieved duty cycle, achieved non-perfused volume and the resulting non-viable tissue volume assessed by histopathology.

Subsequently, the proposed PSA protocol was evaluated in sedated human patients in order to evaluate if the conclusions gained on the animal model with respect to the achieved respiratory depression and the stability of the induced respiratory pattern are transferable into a feasible clinical protocol. For this, two uterine fibroid MR-HIFU treatments and one bone metastasis MR-HIFU treatment were conducted with the proposed PSA protocol and the resulting respiratory pattern was measured directly.

## Methods

### Overall experimental design

The experimental protocol was designed with a clinical MR-HIFU ablation of a small (1–2 cm) malignant liver tumor in mind for which a slow breathing cycle with a long resting phase is required. For this reason, the emphasis was placed on comparing the ablation of the total target volume under PSA with treating a similar target volume under comparable circumstances under GA. In short, two target volumes were planned in the liver at similar shot depths with similar near field tissues and with similar distances to large hepatic vessels. The first target volume was ablated under PSA. This part of the experiment investigated if a remifentanil based PSA protocol can induce a partial respiratory depression, which leads to a slow and regular breathing pattern with long exhalation phases, while maintaining stable and safe vital functions under free-breathing conditions. Subsequently, an adjacent second target volume was ablated under GA with mechanical ventilation. In this part of the experiment, a strong synthetic opiate (sufentanil) induces a complete respiratory depression and the respiratory motion cycle is entirely determined by the parameters of the mechanical ventilation system. For both approaches, a pencil beam navigator on the diaphragm was used for a respiratory-triggered acquisition of the initial anatomical scans used for planning, the PRF-thermometry used for the interventional guidance during the energy deposition and for the acoustic energy deposition itself. Analyses included an evaluation of post-procedural contrast enhanced (CE) T1-weighted images, duty cycle calculations based on the navigator data, MR thermometry data, and histological samples.

### Animal model

The study was approved by our institution’s animal experimental committee (“Dier experiment commissie Utrecht”, reference number 2014.III.01.013, protocol number 104777-1). A porcine model was chosen because of the similarity between the porcine and the human liver in terms of size and perfusion. Furthermore, dosages of remifentanil and propofol reported in literature are comparable for weight-matched pigs and human patients [48, 49]. Healthy female Dalland land pigs of 55–70 kg (– = 8) were placed in prone position on the MR-HIFU system after shaving the skin and application of ultrasound gel.

### Procedural sedation and analgesia and general anesthesia

First, PSA was induced using intravenous (i.v.) infusion of propofol (4.5–6 mg/kg/h) and remifentanil (4.8–5.8 μg/kg/h) by two experienced biotechnicians/veterenarians. Remifentanil has a depressant effect on respiration and a short plasma half-life of <10 min [50]. The depressant effect on respiration allows for a high duty cycle of the HIFU ablation. The short plasma life is favorable for adjusting the breathing frequency and decreases the risk of, e.g., apnea requiring intervention. Volumetric sonications were performed (seven to nine sonications per animal) under PSA. MRI and acoustic energy delivery were respiratory gated using a pencil beam navigator similar to Wijlemans et al. [10]. Subsequently, GA was induced using midazolam (1 mg/kg/h), cisatracurium (0.09 mg/kg/h), and sufentanil (11.3 μg/kg/h). The animal was mechanically ventilated to ensure optimal control over the respiration (Smiths Medical Pneumac paraPAC, Kent, UK). The respiratory frequency was set to 13/min, resulting in a respiratory cycle of 4.5 s with an exhalation phase of 3 s in which the diaphragm is at rest (±1.5 mm). The protocol was repeated after 3 min of cool down to prevent undesired accumulative thermal damage in the near-field. The animal’s blood pressure, exhaled CO_2_ concentration, and heart frequency were monitored during the entire experiment.

### MR-HIFU system

All experiments were performed on a clinical Sonalleve MR-HIFU therapy system (Philips Healthcare, Vantaa, Finland) integrated with a 1.5T Achieva MRI (Philips Healthcare, Best, The Netherlands) [51]. The 256-element HIFU transducer allowed for volumetric ablation using electronic beam steering [52]. An acoustically transparent cooling cushion was placed between the animal and the acoustic window of the HIFU table to cool the skin and minimize the risk of skin burns. Cold water (15 °C) was circulated through the cooling cushion. To prevent systemic hypothermia of the animal, a heating pad with circulating warm water (35 °C) was placed on the back of the animal.

All MR sequences, including planning, thermometry scans, and follow-up contrast-enhanced imaging, were either obtained with respiratory gating or respiratory triggering using 2D navigator pulses (in the scope of this manuscript referred to as *pencil beam navigators* [53]). The pencil beam navigator was placed on the diaphragm adjacent to the ablation area to ensure spatial consistency between all acquired images [53]. Similarly, the energy delivery was respiratory-gated in the following manner: for the end-expiration phase, a gating window was defined with a width of typically 3 mm. The CDAS acquisition system of the MRI was modified to provide a transistor-transistor logic (TTL) signal on a dedicated hardware port, which indicates the presence of the diaphragm in the gating window. Based on this output, an Atmel microcontroller (ATmega32u4, Atmel Corporation, San Jose, California) based interface board generates a 20-Hz trigger signal for the HIFU generator system where a TTL-triggered sonication protocol was executed. Every TTL-pulse triggered a pulse train of 50 ms in length. Arrival of the pulses was logged by the HIFU software and was available for analysis post-treatment.

### MR imaging

Pre-procedural MR imaging included at least one T2-weighted planning scan (3D TSE, TR 1440 ms, TE 130 ms, matrix 208 × 180, FOV 250 × 250 × 200 mm, reconstructed voxel size 0.49 × 0.49 × 3 mm^3^, turbo factor 83). Proton resonance frequency shift (PRFS) thermometry was used for real-time temperature mapping in three planes (GE-EPI, TR 100 ms, TE 15 ms, flip angle 20°, matrix 160 × 160, FOV 400 × 400 mm, voxel size 2.5 × 2.5 × 5 mm^3^, EPI factor 17). One coronal MR slice was centered on the focal point, perpendicular to the symmetry axis of the HIFU beam; one sagittal slice was centered on the focal point, parallel to the beam axis; and one coronal slice was placed in the abdominal muscle for near field monitoring. The pencil beam navigator triggered the acquisition of all three slices at the beginning of the resting phase of the diaphragm. Post-procedural imaging included at least a dynamic contrast-enhanced T1-weighted scan to determine the extent of the non-perfused volume (NPV) (3D gradient echo (THRIVE) TR 5.4 ms, TE 2.6 ms, flip angle 10°, matrix 168 × 132, FOV 80 × 250 × 250 mm^3^, voxel size 0.49 × 0.49 × 3 mm^3^, slice gap −1.5 mm, turbo factor 44, SPIR).

### Ablation procedure

**Fig. 1:**
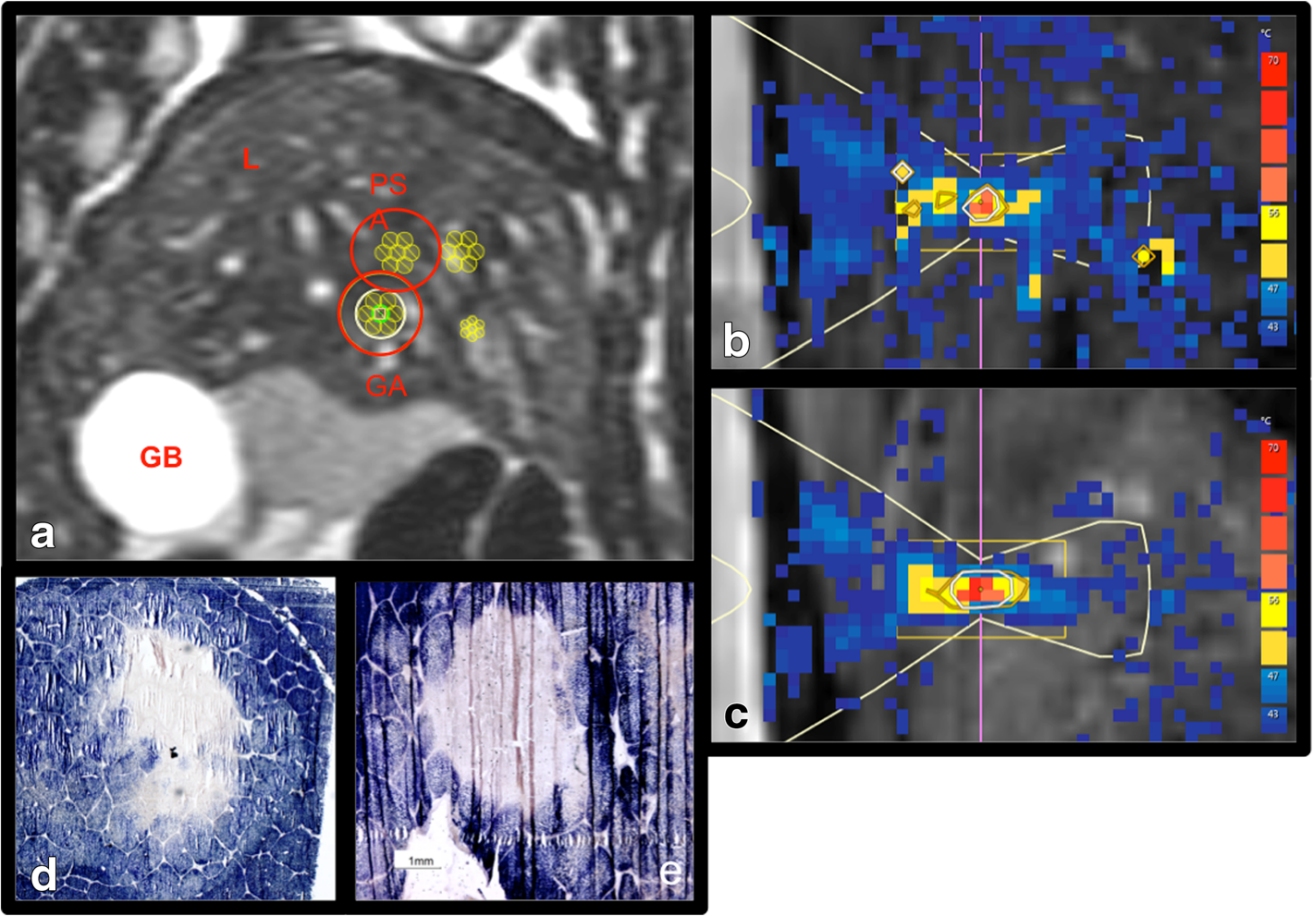
**a** A typical example of shot planning of two clusters of seven treatment cells sonicated under general anesthesia (GA) and procedural sedation and analgesia (PSA) in the liver (L). *GB* gall bladder. **b** A typical example of heating in the focal point under GA in the sagittal plane., whereby the color scale ranges from 43 °C (*blue*) to 70 °C (*red*). **c** A typical example of heating under PSA in the sagittal plane using the same color bar as **b**. **d** Example of nicotinamide adenine dinucleotide (NADH) staining of a lesion ablated under general anesthesia or under procedural sedation and analgesia **e**. Clearly distinguishable regions containing non-viable cells are recognized by its lack of color, while viable cells turn blue after staining

Two target volumes were defined within the liver: one for the ablation under sedation and one as control under general anesthesia. The two chosen parts of the liver were unobstructed by the ribs (inferior to the sternum to avoid excessive heating of the thoracic cage), similar in depth, and similar in distance to large hepatic vessels (Fig. 1). The overall ablation volume was subdivided in small volumetric treatment cells (4 × 4 × 10 mm^3^, 7–10 sonications per target volume), which were sequentially ablated with high acoustic power (450 W acoustic power, 10–25 s per sonication). Acoustic energy delivery was restricted to the resting position of the diaphragm (2–3-mm gating window), while the PRFS thermometry was triggered by the entry of the resting phase. The reference temperature for PRFS thermometry was set to 37 °C. The operator was responsible for aborting the sonication manually when a temperature over 60 °C was observed in the target area of one treatment cell for at least two subsequent temperature images (>6 s). A cool down phase of at least 3 min was allowed between sonications to limit accumulation of heat in the near field.

A post-treatment contrast-enhanced (Gadovist, Bayer, Leverkusen, Germany, 1 mmol/kg) dynamic scan was performed to determine the non-perfused volume (NPV) of both target volumes. After the experiment, the animals were euthanized using an overdose of sodium pentobarbital.

### Histology

The liver was resected on the day of the animal experiment and within an hour after termination of the animal. The coagulated tissue volume, including a margin of healthy liver tissue, was excised and frozen in liquid nitrogen. Frozen samples were prepared for cryosectioning and sliced in 4-μm thick coronal slices each 1000 μm. Slices were stained with hematoxylin and eosin (HE) for general histological assessment and with nicotinamide adenine dinucleotide diaphorase (NADH) histochemistry for cell viability evaluation.

### Patient experiments

Three clinical MR-HIFU treatments of human patients were performed under procedural sedation and analgesia satisfying the required standards and in conformity with regulatory requirements. Two of these interventions were uterine fibroid ablations, one a bone-cancer palliation with HIFU. The conventionally used PSA protocol for these types of intervention at our institution is a combination of ketamine and propofol to induce analgesia and sedation, respectively. Ketamine does not induce respiratory depression, which is for both types of therapy also not required. In the scope of the presented experiments here, the ketamine was replaced by remifentanil.

The first patient was a 57-year-old woman treated for a uterine fibroid. For the induction of the sedation, the following strategy was applied: the remifentanil was kept at a constant dosage, while the propofol dosage was slowly increased to lower the breathing frequency to 5/min. Final dosages for propofol and remifentanil were 1.4 mg/kg/h and 2.5 μg/kg/h, respectively. This stable breathing pattern was reached after <2 h.

The second patient was a 72-year-old woman with bone metastases of which one was located in the shoulder. Here, the strategy was to start with a higher dosage of remifentanil. However, the remifentanil dosage was slowly decreased during the treatment, while the propofol dose was slowly increased to induce a deeper state of sleep. A stable, low frequency (5/min) breathing pattern was reached with 1.6 mg/kg/h propofol and 0.3 μg/kg/h remifentanil in a little under an hour.

The third patient was 41 years old and treated for a uterine fibroid. The dosage of remifentanil was kept constant at 4.2 μg/kg/h and the dosage of propofol was adjusted to the patient’s state of sedation during the treatment and was finally 2.2 mg/kg/h. After the treatment was completed, an additional MR scan (spoiled gradient echo with echo planar imaging, EPI-factor 25, TE 15 ms, TR 39 ms, flip angle 20°, acquisition matrix 120 × 98, pixel size 2.34 × 2.34 mm, slice thickness 7 mm) was performed to visualize the displacement of the diaphragm due to respiration.

One hundred percent oxygen was continuously offered using a nasal prong in all three patients. End tidal carbon dioxide (ETCO_2_) was measured using capnography. Furthermore, breathing frequency, blood pressure values, and heart rate were monitored.

### Data analysis

An image intensity-based semi-automatic segmentation algorithm (MeVisLab, MeVis Medical Solutions AG, Bremen, Germany) [4] was used to determine the NPVs on CE-T1w imaging to compare the lesions ablated under PSA and GA. An NPV per sonication was calculated by dividing the total NPV of each lesion by the number of treatment cells composing that particular lesion. Wilcoxon signed-rank test was used to test for equality in NPV.

Areas of non-viable tissue were calculated by measuring the width and the length of the region showing less or no staining NADH histology sections and assuming an ellipsoidal shape. The distance between two subsequent sections was 1000 μm. A total non-viable lesion volume was calculated by summing the volumes of the individual sections. Wilcoxon signed rank test was used to check for equality in necrosed tissue volume.

Duty cycles (ratio or percentage of the breathing cycle with the diaphragm in its resting position, such that acoustic energy delivery with MR-HIFU was allowed) were calculated based on the logged TTL-pulses arriving at the HIFU generator system as a result of the previously described logic. The duty-cycle (*dc*) was calculated as follows

$$ dc=\frac{n_{\mathrm{pulse}}T}{t_{\mathrm{last}}-{t}_{\mathrm{first}}} $$

where *n*_pulse_ is the number of pulses that trigger energy delivery, *T* the length of a single pulse-train (=50 ms), *t*_first_ (ms), and *t*_last_ (ms) are the arrival time of the first and last pulse triggering energy delivery.

Both the duration of the sonication and the duty cycles during the entire ablation of one target volume were compared for PSA and GA using the Student’s *t* test after verifying normality using P-P plots.

To visualize the breathing pattern in a patient under sedation a vertical line through the diaphragm on the MR image was plotted over time using all the dynamics of the scan.

## Results

### Non-perfused volume

**Fig. 2:**
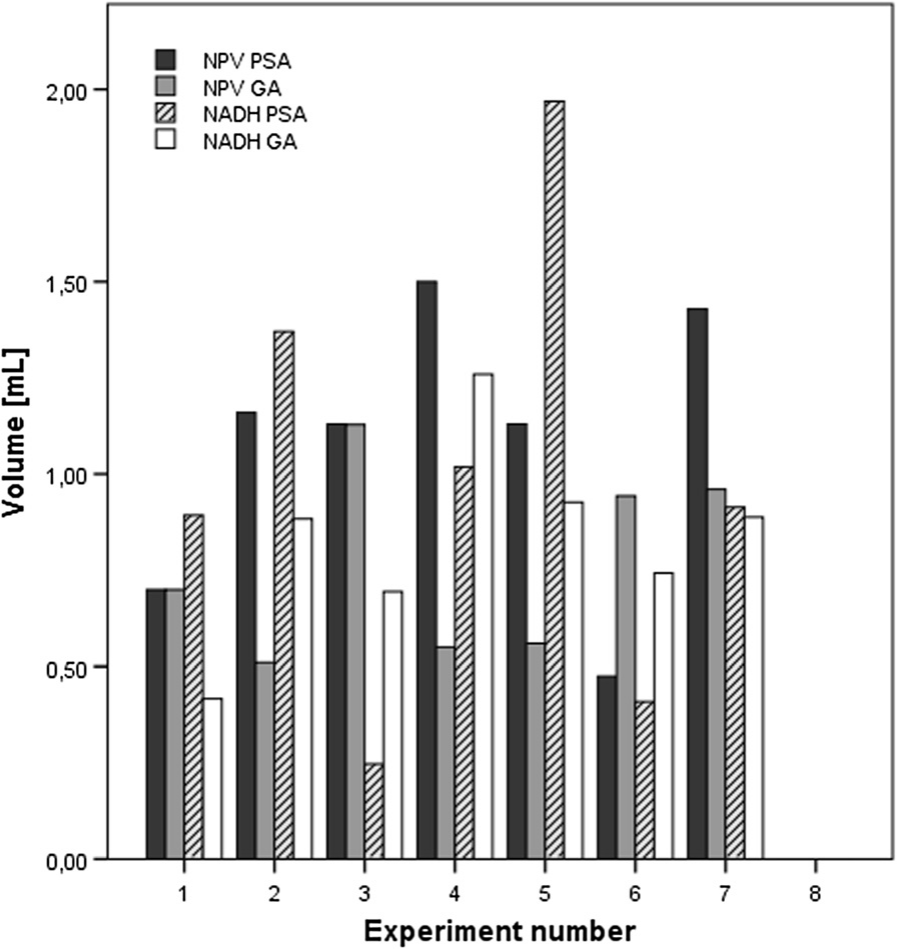
Ablation volumes per sonication for procedural sedation and analgesia (PSA) and general anesthesia (GA). *NPV* ablation volumes based on the non-perfused volume (NPV) as obtained from contrast-enhanced MR-scans, *NADH* non-viable tissue volumes based on nicotinamide adenine dinucleotide (NADH) staining, *TD* ablation volume based on the lethal thermal dose (TD) volume as calculated by the HIFU software

A total of 57 sonications were performed under PSA in eight animals with a median of seven sonications per target volume (range seven to eight sonications per target volume). A total of 61 treatment cells were ablated under GA in the same eight pigs with a median of 7 (range 7–10 treatment cells per target volume). The non-perfused volume created in one pig could not be analyzed as sonications overlapped with those of a preceding experiment. The sonications resulted in a median NPV of 1.16 mL (IQR 0.70–1.31 mL, range 0.48–1.50 ml) for the target volumes ablated under PSA and 0.70 mL (IQR 0.55–0.96 ml, range 0.51–1.29 ml) under GA (Table 1 and Fig. 2). The average NPV per sonication was 0.15 mL (range 0.07–0.21 mL/sonication) for ablations under PSA and 0.10 mL (0.057–0.18 mL/sonication) under GA. Wilcoxon signed-rank test showed that the NPV created under PSA and GA is statistically not different (*p* = 0.173).

**Table 1:** Overview of the number of sonications performed to create a lesion per experiment performed either under procedural sedation and analgesia (PSA) or general anesthesia (GA), of the non-perfused volume (NPV) obtained from these sonications, the mean NPV created per sonication, the non-viable volume as observed by nicotinaminde adenine dinucleotide (NADH) staining for the entire cluster, and the mean non-viable volume per sonication

Experiment	#sonications		NPV (mL)		NPV/sonication (mL)	
	PSA	GA	PSA	GA	PSA	GA
1	7	10	0.70	0.70	0.10	0.070
2	8	9	1.16	0.51	0.15	0.057
3	7	7	1.13	1.29	0.16	0.18
4	7	7	1.50	0.55	0.21	0.079
5	7	7	1.31	0.56	0.19	0.080
6	7	7	–	–	–	–
7	7	7	0.48	0.94	0.069	0.13
8	7	7	1.30	0.96	0.19	0.14
Experiment	NADH (mL)		NADH/sonication (mL)			
	PSA	GA	PSA	GA		
1	0.89	0.46	0.13	0.046		
2	0.95	0.19	0.12	0.021		
3	0.13	0.46	0.019	0.066		
4	1.01	0.05	0.14	0.0071		
5	1.55	0.47	0.22	0.067		
6	–	–	–	–		
7	0.41	0.74	0.056	0.11		
8	0.92	0.89	0.13	0.13

### Non-viable tissue volume

Sectioning and NADH staining of the excised liver tissue volumes showed non-viable tissue (recognizable by its lack of staining), a border zone (recognizable by its pale blue color, however, not observed in all cases), and viable tissue, which appears as dark blue due to the stained samples (Fig. 1). Both the non-viable region and the border zone were calculated as non-viable tissue, since it was reported previously that the necrotic tissue area after MR-HIFU ablation increases during the first day post treatment and analyses performed in the first few hours lead to an underestimation of the necrotic tissue volume [54–56]. Examples of sections with clearly distinguishable non-viable and viable regions obtained under PSA or GA are shown in Fig. 1.

Lengths and widths were measured and volumes were calculated assuming an ellipsoidal area and a slice thickness of 1000 μm. Under PSA, a median non-viable tissue volume of 0.92 mL (IQR 0.41–1.37 mL, range 0.25–1.37 mL) was ablated. The median non-viable tissue volume obtained under GA was 0.88 mL (IQR 0.70–0.93, range 0.46–1.26 mL). Per sonication a median non-viable tissue volume of 0.12 mL (range 0.06–0.28 mL/sonication) was obtained under PSA. The median non-viable tissue volume obtained under GA was 0.13 mL per sonication (range 0.066–0.18 mL/sonication). Wilcoxon signed-rank test proved that there was no statistically significant difference in necrosed tissue volume between PSA and GA.

While excising the ablated liver volumes, the near field and the skin were carefully checked for damage and skin burns. No abnormalities were observed in the skin, subcutaneous fat layer or abdominal muscle in all cases. Also Glisson’s capsule of the liver was intact in all eight pigs, even when the ablation zone extended to the outer surface of the liver.

### Breathing frequency, duty cycle, and the duration of the sonications

The breathing frequency was fixed at 13/min under general anesthesia by using mechanical ventilation. Stable free breathing patterns were obtained under PSA. Breathing frequencies under PSA varied between 9 and 15 breaths per minute between the animals and during the experiment. Steady-state is reached after at least 20 min depending on the desired decrease in breathing frequency.

Under PSA a median duty cycle of 79.5 % (IQR 72.5–84.5, range 18.0–96.0) was achieved, while under GA this was only 64.0 % (IQR 62.0–66.5, range 46.0–73.0). These values differed significantly (*p* < 0.001).

**Fig. 3:**
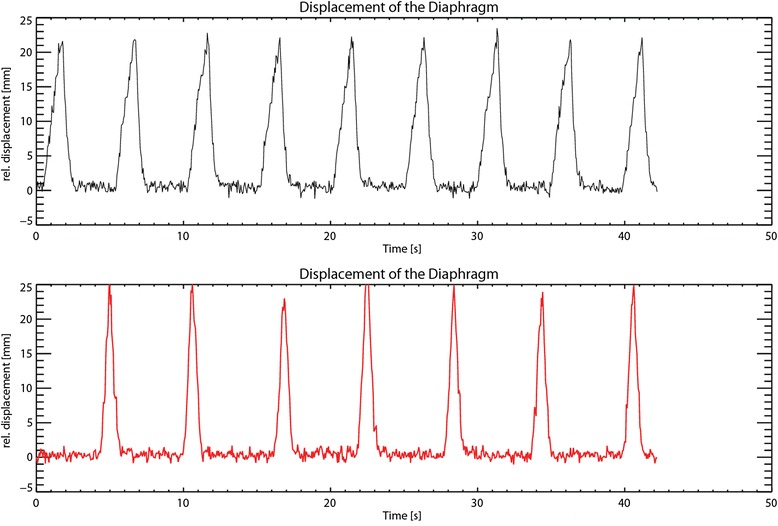
Displacement of the diaphragm as measured with MRI. *Top*: general anesthesia (GA). *Bottom*: procedural sedation and analgesia (PSA). The displacement of the diaphragm is in both cases less than 3 mm during the resting phase causing only a small heat-spread during ablation. The resting phase is longer under PSA, which is due to the dosages of remifentanil and propofol and the settings of the mechanical ventilator used under GA

Figure 3 shows the displacement of the diaphragm under PSA and under GA. In the resting phase, the displacement was limited to three millimeters for both anesthesia protocols.

The duration per sonication was recorded (*n* = 57 for PSA, *n* = 61 for GA). P-P plots showed a normal distribution for the PSA group and the GA group. A Student’s *t* test showed that the duration of the sonications performed under PSA was significantly shorter (*p* = 0.000) with mean values of 15.0 s (range 6.70–25.70 s) for PSA and 20.3 s (range 10.0–27.0 s) for GA. This is in agreement with the difference in duty cycle found between both groups.

### Monitoring of vital functions

The percentage of CO_2_ (end-tidal: ETCO2) in the expired air remained below 5.5 % during the entire experiment performed under sedation in all five pigs. Blood pressure and heart rate values remained within the normal range during all sonications under PSA in all pigs. Apnea was observed in one pig while the correct dosages for remifentanil and propofol were set by the veterinarian and biotechnicians to obtain a stable state of sedation and respiration. Respiration was quickly restored by lowering the dosage of remifentanil and by inducing several inhalations manually with a self-inflating bag. Under GA, all vital functions were stable and within the standard range. No abnormalities were observed.

### Patient treatments

**Fig. 4:**
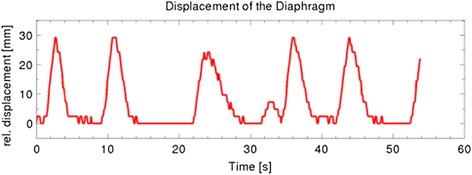
Displacement of the diaphragm in a patient under procedural sedation and analgesia measured with MRI. Resting phases of 5–10 s can be observed

During all three patient treatments under PSA, the breathing cycle could be depressed to values as low as 5 per minute with a rapid inhalation and exhalation phase and a long resting phase as visualized by capnography. The ETCO_2_ level stayed below 6.5 %. Blood pressure and heart rate values remained within the normal range indicating the absence of pain sensation that can be caused by HIFU. The displacement of the diaphragm over time is shown in Fig. 4. In the presented case, the maximum displacement is 3 cm after inhalation. A resting phase could be observed of 5–10 s between breaths, which was found similar in all three patients. Particularly noteworthy is the change of the diaphragm kinetics from a smooth inhalation/exhalation event under non-depressed free-breathing to a breathing pattern with a short and rapid gas exchange, followed by long plateau of immobility of the diaphragm.

## Discussion

Previous work demonstrated that respiratory-gated HIFU energy delivery and MR-guidance can achieve confluent treatment volumes in porcine liver tissue within a clinically accepted time frame under the conditions of general anesthesia [10]. In this study, we investigated if comparable results can be achieved by releasing the requirement for general anesthesia and instead delivering acoustic energy during a partially depressed but free-breathing motion pattern, which results from a tailored procedural sedation and analgesia protocol. PSA is known to cause fewer complications, to provide for a faster recovery after treatment, and to decrease the burden for the patient, without hampering the MR-HIFU treatment.

The data of the pencil beam navigator, which tracked the displacement of the diaphragm over time directly, displays minimal displacement during the resting phase of the breathing cycle under PSA. Over the duration of 5–7 s, the diaphragm was found virtually completely static, allowing long periods of energy delivery by MR-HIFU to the target volume without any requirement of sophisticated motion correction. Free breathing patterns without respiratory depression show after the initial rapid inhalation event typically long transitional phases between inhalation and exhalation, during which the diaphragm is slowly moving [43]. Moreover, typical repertory motion patterns under free-breathing are only periodical over short epochs of 20–30 s, before the pattern changes or is interrupted by spontaneous motion events. Both characteristics were entirely suppressed by the remifentanil-propofol protocol used in this study. The respiratory pattern has been found to be very regular and free of spontaneous breathing events over epochs of 1 h without visible modifications to the pattern, nor a measurable degradation of the vital functions of the animal. Furthermore, this breathing pattern was found reproducible in all animals and little variation was observed inter-individually. This is of significant clinical value, as PSA protocols need to be reliable and reproducible for patient care.

The resulting breathing pattern with long static phases of the diaphragm obtained under PSA with remifentanil and propofol results in similar NPVs and non-viable volumes (NADH staining) as published previously [10] and as shown in this study acquired under GA. The lesions ablated under PSA were confluent and created at the intended location in the liver. These effects were reproducible, since they were observed in all seven animals.

Although NADH staining is a reliable indicator of mitochondrial activity and thus cell viability, its sensitivity is limited within the first hours after HIFU ablation, since it takes only acute cell death into account and not the result of apoptosis. This often results in an under-estimation of the necrotic tissue area [55, 56] and a mismatch with the NPV, which is typically found larger. Since the animals were sacrificed at the end of the experiments, any increase of the necrotic volume over time could not be measured. This effect has been observed previously for MR-HIFU of the liver [10]. Also in this study, the mismatch between the NPV and non-viable NADH volume has been observed, especially for the GA ablation volumes, since these were performed after the PSA ablations and thus sooner upon termination of the animal.

NPV volumes per sonication were higher for ablations performed under sedation than for sonications under GA. Also, the non-viable volumes obtained by NADH staining of the tissue sections showed larger necrotic volumes for PSA than for GA. These values are influenced by the duty cycle and thus by the breathing frequency. The breathing frequency was set to a fixed value under GA in order to maintain comparability to previous work on MR-HIFU of the liver [10] and was lower under PSA. Similar results for PSA and GA can be expected if the breathing frequency of the GA protocol would have been matched to the results obtained during PSA on an inter-individual basis. Therefore, the results obtained under PSA have not to be considered superior to GA, but we can conclude that essentially equivalent results can be achieved with either method.

More important is the displacement of the diaphragm, which is an estimate of the displacement of the liver. Large displacements during sonications cause heat-spread. The consequence of this effect is twofold: (1) The temperature in the target region may not reach 56 °C resulting in damage but not in complete cell death in the ablation area, which has major consequences when treating malignant tumors and (2). Healthy tissue might be damaged, which is undesirable. We have shown in this study that the residual motion of the diaphragm in the resting phase of the breathing cycle is <3 mm both under PSA and under GA. This is very small compared to the displacement during inhalation and exhalation, which is 20–25 mm, as is shown in Fig. 3, and was found acceptable for accurate energy delivery as is previously shown by our group using the same tidal volume [10].

The decrease in breathing frequency and the long resting phase of the diaphragm are specific characteristics of remifentanil usage. Remifentanil is a very potent opioid and is metabolized by non-specific esterases to a much less potent metabolite [57]. Apart from its depressant effect on the respiratory cycle, it has other advantages. It has rapid onset of action (blood-brain equilibration time of 1 min) [58], as well as a short half-life time ranging between 3 and 5 min, which is independent of the duration of the infusion [49]. Remifentanil allows for a rapid transition from intense analgesia to minimal residual effect [49]. Recovery from PSA with remifentanil is therefore very quick, which is beneficial for clinical practice, in particular considering elderly and/or fragile patients. Additionally, remifentanil has a strong analgesic effect that can alleviate symptoms of discomfort caused by the treatment.

Propofol is a widely used hypnotic/amnestic drug for sedation during a broad range of interventional procedures [49, 59]. It has several advantages, such as rapid onset of the sedative effect (approximately 30 s), a short action time (distribution half-life 2–4 s and elimination half-life 30–60 s), and a rapid and complete recovery profile, and it induces both sedation and amnesia, which is favorable to the patient considering the duration of MR-HIFU procedures [60, 61]. However, propofol lacks analgesic effects and does not induce respiratory depression. Therefore, the combination of remifentanil and propofol appears complementary. Other advantages of this combination are the decrease in pain arising from propofol injection by remifentanil, the decrease in nausea and vomiting due to opioids by propofol, the reduced required dose of both medications when administered together, and resulting accelerated recovery [62].

However, as we observed in this study, the induction of a partial respiratory depression inherently introduces the risk of respiratory arrest due to remifentanil over dosage. Fortunately, in this case, the respiratory cycle can be quickly restored to a stable pattern by decreasing the dosage due to the high clearance rate, while providing mechanically assisted ventilation. When inducing sedation with remifentanil, the starting dose should be in the lower range of known effective dosages and the dosages of both remifentanil and propofol should be attuned during the preparation phase of the treatment to find the ideal state, which is stable and induces the desired breathing frequency. Vital functions should be monitored closely during this phase and should stay within the standard range (ETCO_2_: 36 ± 5 mmHg or ± 5 % [63–65]). Nevertheless, the dosages administered during all three MR-HIFU procedures in patients were comparable to the values of clinically established sedation protocols for, e.g., endoscopy procedures under remifentanil and propofol (0.05 μg/kg body weight/min remifentanil and 50 μg/kg body weight/min propofol [49]) and are thus in their added risks and the required emergency procedures well understood.

On the other hand, the supporting veterinary department did not have an established protocol for PSA with remifentanil and propofol for treatments requiring partial respiratory depression in a porcine model. Therefore, the exact protocol, in particular the dose escalation of each drug had to be developed in the scope of this study in the preparation of each treatment. In particular, in the initial phase of the study (*n* = 1, 2), this did lead to a prolonged preparation until the animals reached a stable breathing pattern. For later experiments (*n* = 3–8), the duration between the induction of PSA until a stable breathing pattern was reached required ~20 min. In comparison, dosages used for GA during the porcine experiments are well known and also the settings of the mechanical ventilator at 13 breaths per minute with a volume of 0.4 L per breath have been used many times before during porcine MR-HIFU experiments [10].

## Conclusions

In the first part of this study, we demonstrated the feasibility and reproducibility of MR-HIFU ablations of the liver under remifentanil and propofol induced PSA in a porcine model. We have shown that partial respiratory depression resulting in a long resting phase of the diaphragm and high duty cycles of acoustic energy delivery is feasible. The clinical application of this sedation protocol would be MR-HIFU treatments of small primary or metastatic liver tumors in human patients. Therefore, we explored in the second part of this study in three patients treated with MR-HIFU (uterine fibroid and a bone metastasis) if the main findings, in particular the possibility to reduce the breathing frequency to 5/min, while obtaining a long exhalation period in which the diaphragm is stationary, are transferable from the porcine model to patients. We confirmed the low breathing frequency and high duty cycle desired for successful MR-HIFU treatments in three patients using the same combination of medication and while maintaining stable vital functions. Furthermore, we showed that a resting phase of the diaphragm of 5–10 s could be induced under PSA in one patient. In conclusion, we propose the combination of remifentanil and propofol during MR-HIFU as PSA is less invasive and carries fewer risks for the patients while showing equal results when compared to GA.

## Abbreviations

CE, contrast enhanced; ETCO_2_, end-tidal carbon dioxide; FOV, field of view; GA, general anesthesia; GE-EPI, gradient echo echo planar imaging; HE, hematoxylin and eosin; HIFU, high-intensity focused ultrasound; IQR, inter quartile range; MR, magnetic resonance; NADH, nicotinamide adenine dinucleotide diaphorase (reduced form); NPV, non-perfused volume; PRF, proton resonance frequency; PSA, procedural sedation and analgesia; TE, echo time; TR, repetition time; TSE, turbo spin echo; TTL, transistor-transistor logic
